# Case report. Necrose van de glans penis na prostaatembolisatie

**DOI:** 10.1007/s13629-022-00373-y

**Published:** 2022-12-22

**Authors:** Lien Vanderlinden, Rob J. A. M. Davits

**Affiliations:** Elisabeth-Tweesteden Ziekenhuis, Tilburg, Nederland

**Keywords:** embolisatie, prostaat, necrose, ischemie, complicatie, embolisation, prostate, necrosis

## Abstract

Prostaatembolisatie is een nieuwe minimaal-invasieve procedure die onder andere wordt ingezet bij benigne prostaathyperplasie. Bij nieuwe technieken komen echter ook nieuwe complicaties kijken. In deze uitgebreide case report bespreken we vier patiënten bij wie necrose van de glans penis optrad na de embolisatie van de prostaat. De oorzaak van deze complicatie is niet bij alle patiënten duidelijk, en meer onderzoek is zeker nodig. Patiënten dienen op de hoogte gesteld te worden van deze complicatie, gezien de soms zeer uitgebreide esthetische veranderingen die embolisatie teweeg kan brengen.

## Abstract (en)

Prostate artery embolization is a new minimal invasive technic to combat benign prostate hyperplasia. This new technic also comes with new complications. In this case report, we look at four patients in whom a necrosis of the glans penis occurred after the embolization of their prostate arteries. The cause of this complication is not yet clear in all of the patients and further research is still necessary. Patients who are considering an embolization of their prostate arteries need to be informed about this complication because of the drastic aesthetic outcomes that can sometimes be seen.

## Introductie

Prostaatembolisatie is een relatief nieuwe techniek bij benigne prostaathyperplasie (BPH) en -bloedingen. Bij deze minimaal-invasieve procedure wordt de arteria prostatica opgezocht via de arteria femoralis communis in de lies en worden HydroPearl®-microsferen ingespoten.

Embolisatie blijkt een effectieve methode voor het verhelpen van plasklachten [1]. Ook in spoedsituaties blijkt het emboliseren van de prostaat met hematurie op basis van BPH effectief [2]. Voor andere mogelijke toepassingen wordt embolisatie nog verkend, bijvoorbeeld bij laaggradig prostaatkanker [3].

Een van de meest gevreesde complicaties van embolisatie is ischemie van de penis en dan met name van de glans. De arteria pudenda interna is de gemeenschappelijke oorsprong van zowel de peniele arteriën als de arteria prostatica. Bij de embolisatie bestaat dus de mogelijkheid dat microsferen ontsnappen naar de verkeerde takken. Daarnaast bestaan bij 57% van de mannen, als anatomische variant van het vaatstelsel, intraprostatische peniele collateralen, waardoor microsferen, ondanks alle voorzorgsmaatregelen, in de peniele arteriën terechtkomen [4].

In het ETZ zijn, sinds 2011, 122 embolisaties uitgevoerd bij 102 patiënten vanwege LUTS of hematurie op basis van BPH. Bij twee patiënten werd een milde hypoxie van de glans gezien; bij vier patiënten een necrose. Van deze vier patiënten doen wij hier verslag.

## Casus 1

Een 81-jarige man presenteerde zich initieel met pollakisurie en urgencyklachten. Met een prostaat van 164 cc werd eerst getracht met dutasteride/tamsulosine de klachten onder controle te krijgen. Uiteindelijk werd voor embolisatie gekozen, waarbij beiderzijds protection coils geplaatst werden wegens collateralen naar de arteria dorsalis penis. Rechts werden 400 en 600 µm Hydropearl®-microsferen gebruikt, links enkel 400. De procedure verliep moeizaam, maar wel ongecompliceerd.

**Figure 1:**
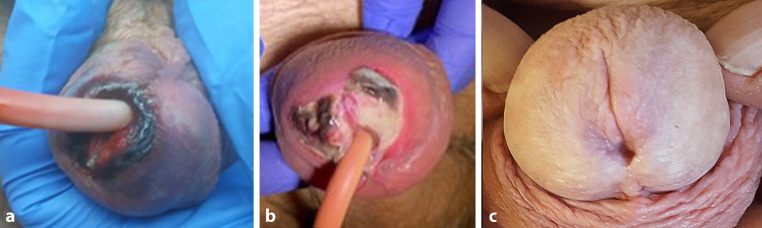

17 dagen na de embolisatie presenteerde de patiënt zich met een hypoxie rondom de meatus (fig. 1a). Initieel werd er gedacht aan decubitus bij de katheter. Bij verdere observatie werden de eerste tekenen van necrose gezien (fig. 1b). De situatie werd conservatief opgevolgd. Na iets meer dan een maand werd een spontane genezing van de glans gezien (fig. 1c). De patiënt zelf ondervond pijnklachten zodra de necrose ontstond, maar was tevreden met het eindresultaat van de wond. Meneer bemerkte na de embolisatie een duidelijke verbetering in de mictie.

## Casus 2

Een 72-jarige man, met in de voorgeschiedenis een laser-TURP, presenteerde zich met terugkerende hematurie op basis van prostaatbloedingen en was transfusiebehoeftig. Er werd een spoedembolisatie van de prostaat uitgevoerd. Tijdens de procedure werden fijne collateralen naar de anusregio beschreven. Er werden zowel HydroPearl®-microsferen van 200 µm als van 400 µm gebruikt. Tijdens de procedure werd reflux gezien richting de arteria dorsalis pedis rechts. De volgende dag begon een lichte paarsverkleuring van de glans, die de dagen erna verergerde.

**Figure 2:**
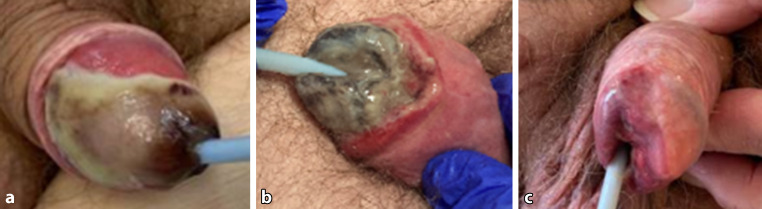

13 dagen na de embolisatie vond heropname plaats vanwege demarcatie van de glans (fig. 2a). De necrose nam verder toe tot 30 dagen na de procedure (fig. 2b). Behandeling bleef conservatief. Er waren niet veel pijnklachten. 50 dagen na de embolisatie resteerde een hypospadie (fig. 2c); de patiënt was zelf tevreden met het resultaat, al blijft hij last houden van sproeiende mictie. Na de embolisatie bleef hematurie uit. De katheter kon na genezing van de wond verwijderd worden.

## Casus 3

Een 62-jarige patiënt presenteerde zich met LUTS bij een prostaatvolume van 66,8 cc. Ook bij deze patiënt werd primair met dutasteride/tamsulosine gestart zonder goed resultaat. Omdat door COVID-19 plannen van overige operaties niet op korte termijn mogelijk was, werd in overleg met de patiënt gekozen voor prostaatembolisatie. Tijdens de procedure werden links zowel een *protection coil* in de arteria pudenda geplaatst richting de arteria dorsalis pedis als twee occlusieballonnen. Er werden 200 en 400 µm HydroPearl®-microsferen gebruikt. Rechts verliep de procedure zonder complicaties en zonder protectie.

**Figure 3:**
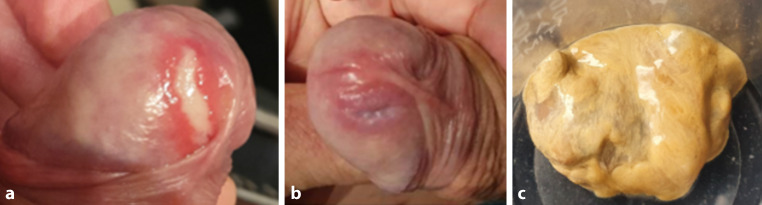

12 dagen na de embolisatie werd een klein necrotisch wondje gezien (fig. 3a). Zes weken na de embolisatie was dit wondje volledig genezen met achterlating van een litteken (fig. 3b). Er werd geen transurethrale katheter ingebracht; na het uitplassen van enkele grote stukken prostaat (fig. 3c) was de patiënt enorm tevreden met de mictie.

## Casus 4

Een 73-jarige patiënt presenteerde zich met LUTS bij een prostaatvolume van 140 cc. Na analyse van de plasklachten werd gezamenlijk gekozen voor prostaatembolisatie. De procedure verliep zonder bijzonderheden. Er werden 200 en 400 µm HydroPearl®-microsferen gebruikt. *Protection coils* waren niet van toepassing.

**Figure 4:**
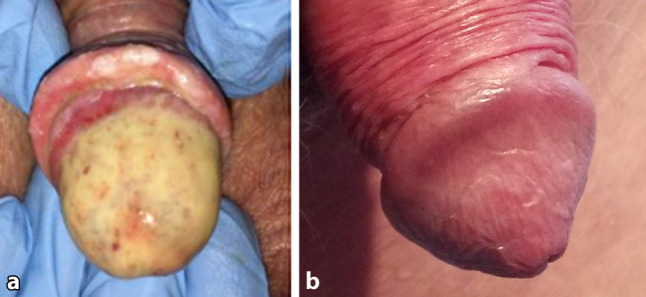

Vier dagen na de embolisatie trad lichte paarsverkleuring van de glans op. Na weer acht dagen was nagenoeg de hele glans genecrotiseerd en was de voorhuid nagenoeg volledig verloren gegaan (fig. 4a). De patiënt had hierbij zeer hevig pijnklachten. Na enkele weken trad spontane genezing op (fig. 4b). De pijn nam af en uiteindelijk was er een esthetisch mooi resultaat. De patiënt is tevreden met de mictieresultaten.

## Discussie

Necrose van de glans penis na prostaatembolisatie is voorlopig een moeilijk te voorspellen complicatie. Slechts bij één van de vier patiënten werd peroperatief een duidelijke oorzaak gevonden voor de necrose. Er wordt gedacht dat de HydroPearl®-microsferen van 200 µm te klein zijn, waardoor *protection coils* niet altijd voldoende effect hebben. Ook de coil zelf zou een probleem kunnen vormen, maar dergelijke coils worden echter veel vaker gebruikt dan dat er complicaties voorkomen, wat deze theorie weerlegt. Er zijn studies waarin is aangetoond dat een coil veilig gebruikt kan worden om juist deze non-target embolisatie te voorkomen [5]. Mogelijk zijn collateralen vanuit de prostaat de reden dat bij drie van deze vier patiënten sprake was van deze complicaties, maar om dit aan te tonen, zal verder onderzoek nodig zijn. Het feit dat we deze complicaties niet terugvinden bij onder andere een prostatectomie weerlegt deze theorie toch in enige mate.

Omdat prostaatembolisatie nog een experimentele behandeling is, weten we nog niet zo veel over de epidemiologie van complicaties zoals necrose van de glans penis. Als we naar de cijfers kijken in onze kliniek is er 3% kans op necrose. In werkelijkheid zal deze kans waarschijnlijk groter zijn; voordat we door enkele extreme gevallen getriggerd werden, zijn waarschijnlijk klachten van de glans penis vaak alleen aan een eventuele transurethrale katheter toegeschreven. Deze complicatie verdient dus zeker een plaats bij het informeren van de patiënt over de procedure. Uiteindelijk lijkt er een spontaan herstel plaats te vinden en waren deze patiënten tevreden.

## Conclusie

Embolisatie van de prostaat is een nieuwe techniek, onder andere ter behandeling van plasklachten als gevolg van BPH. De eerste resultaten lijken veelbelovend. Er zijn echter ook onverwachte complicaties. Bij de voorlichting van de patiënt is vermelding van mogelijke necrose van de glans penis dan ook belangrijk. Wie onbekend is met deze complicatie loopt het risico necroseklachten toe te schrijven aan een eventueel aanwezige transurethrale katheter.
